# Lean Body Mass Predicts Long-Term Survival in Chinese Patients on Peritoneal Dialysis

**DOI:** 10.1371/journal.pone.0054976

**Published:** 2013-01-25

**Authors:** Jenq-Wen Huang, Yu-Chung Lien, Hon-Yen Wu, Chung-Jen Yen, Chun-Chun Pan, Tsai-Wei Hung, Chi-Ting Su, Chih-Kang Chiang, Hui-Teng Cheng, Kuan-Yu Hung

**Affiliations:** 1 Department of Internal Medicine, National Taiwan University College of Medicine and Hospital, Taipei, Taiwan; 2 Department of Integrated Diagnostics and Therapeutics, National Taiwan University College of Medicine and Hospital, Taipei, Taiwan; 3 Department of Nursing, National Taiwan University College of Medicine and Hospital, Taipei, Taiwan; 4 Department of Internal Medicine, Buddhist Tzu Chi General Hospital, Taipei Branch, New Taipei City, Taiwan; 5 Department of Internal Medicine, Far Eastern Memorial Hospital, New Taipei City, Taiwan; 6 Department of Internal Medicine, National Taiwan University College of Medicine and Hospital, Yun-Lin Branch, Yun-Lin County, Taiwan; 7 Department of Internal Medicine, National Taiwan University College of Medicine and Hospital, Hsin-Chu Branch, Hsin Chu City, Taiwan; University of Sao Paulo Medical School, Brazil

## Abstract

**Background:**

Reduced lean body mass (LBM) is one of the main indicators in malnutrition inflammation syndrome among patients on dialysis. However, the influence of LBM on peritoneal dialysis (PD) patients’ outcomes and the factors related to increasing LBM are seldom reported.

**Methods:**

We enrolled 103 incident PD patients between 2002 and 2003, and followed them until December 2011. Clinical characteristics, PD-associated parameters, residual renal function, and serum chemistry profiles of each patient were collected at 1 month and 1 year after initiating PD. LBM was estimated using creatinine index corrected with body weight. Multiple linear regression analysis, Kaplan–Meier survival analysis, and Cox regression proportional hazard analysis were used to define independent variables and compare survival between groups.

**Results:**

Using the median LBM value (70% for men and 64% for women), patients were divided into group 1 (n = 52; low LBM) and group 2 (n = 51; high LBM). Group 1 patients had higher rates of peritonitis (1.6 vs. 1.1/100 patient months; p<0.05) and hospitalization (14.6 vs. 9.7/100 patient months; p<0.05). Group 1 patients also had shorter overall survival and technique survival (p<0.01). Each percentage point increase in LBM reduced the hazard ratio for mortality by 8% after adjustment for diabetes, age, sex, and body mass index (BMI). Changes in residual renal function and protein catabolic rate were independently associated with changes in LBM in the first year of PD.

**Conclusions:**

LBM serves as a good parameter in addition to BMI to predict the survival of patients on PD. Preserving residual renal function and increasing protein intake can increase LBM.

## Introduction

Protein–energy wasting (PEW) presents as low serum albumin and serum cholesterol levels, low body mass index (BMI), and reduced dietary protein intake [1]. In the general population, this condition is often associated with metabolic stresses and an inadequate diet. However, in patients with chronic kidney disease (CKD), loss of lean body mass (LBM) relates to reduced nutrient intake [1] and consistently high mortality [2], [3]. In patients on hemodialysis (HD), lower LBM negatively influences survival, as does age [4]. Other studies that followed patients up to 20 more months also showed that LBM predicted survival among PD patients [5]–[7], indicating the importance of LBM in this population. However, the factors affecting LBM changes remain unclear.

LBM can be measured using the creatinine index derived from creatinine kinetics. Creatinine clearance from dialysate and urine, in addition to creatinine degradation, represent patient dietary skeletal muscle protein intake and muscle mass [8]. The creatinine index can be used to accurately estimate fat-free body mass in dialysis patients [6], [9], [10], [11].

The current study enrolled incident PD patients and measured their LBM at 1 month and 1 year after initiating PD, then followed their clinical outcomes for >8 years. The aims of this study were to investigate the impact of LBM on patient outcomes and the factors that are associated with LBM changes. This study demonstrates that LBM significantly affects PD patient survival and establishes the factors that may increase LBM.

## Methods

### Patients

Patients who started PD as a chronic renal replacement therapy between January 2002 and December 2003 were enrolled in this study. PD clearance and residual renal function were measured 1 month and 1 year after PD initiation. Follow-up continued until December 2011.

### Clinical Characteristics and Follow-up

Clinical characteristics and dialysis parameters were reviewed from the medical records and included body mass index (BMI), peritoneal equilibration test (PET) results, adequacy Kt/V, residual renal function (renal KT/V), and normalized protein catabolic rate (nPCR). The results of regular serum chemistry studies including blood urea nitrogen (BUN), creatinine, albumin, total cholesterol (CHO), and triglycerides (TG), and total iron binding capacity (TIBC) were also reviewed. These data were collected at the initial evaluation 1 month and 1 year after PD initiation. LBM was evaluated using the creatinine index at these 2 time points. The change between the data at 1 month and at 1 year was calculated using the following formula:

change in LBM = 100 × (LBM_1y_ – LBM_1m_)/LBM_1m._

After initiation of PD, patients were followed prospectively for the occurrence of hospitalization, peritonitis, technique failure, and mortality. Patients who received transplants were censored in assessments of technique failure and mortality rates.

### Ethical Considerations

All medical records and individual laboratory data were reviewed in this study. The study was also approved by the ethics committee of National Taiwan University Hospital under NTUH-REC No. 201205010RIC.

### Calculation of Creatinine Index and Lean Body Mass

The creatinine index is measured as the sum of creatinine removed from the body (measured as the creatinine removed in dialysate, ultrafiltrate, and urine), any increase in the body creatinine pool, and the creatinine degradation rate [11]. This study assumed that the creatinine levels in patients on PD were stable, so no change in the body creatinine pool was included in the creatinine index calculation. Thus, the creatinine index was simplified to the formula [12]: 

Creatinine index (mg/24 h) = Effluent creatinine+urine creatinine) (mg/24 h)+creatinine degradation (mg/24 h).

Creatinine degradation was further estimated using the following equation: 

Creatinine degradation (mg/24 h) = 0.38 dL/kg/24 h × serum creatinine (mg/dL) × body weight (kg).

The creatinine index can be used to estimate edema-free LBM using the equation [13]:

Edema-free lean body mass (kg) = (0.029 kg/mg/24 h) × creatinine index (mg/24 h) +7.38 kg.

The calculated LBM corrected with individual body weight (BW) as the percentage of BW.

### Statistical Analysis

All variables are reported as mean ± SD (or with 95% confidence intervals where appropriate) for continuous variables and as frequencies or percentages for categorical variables. Student’s *t* test was used for analysis between groups where appropriate. Differences in frequency were tested using Chi square analysis. Relationships between variables were tested using Pearson correlation. The independent determinants of any variable were analyzed using multiple linear regression analysis. The adjusted variables were stated in each analysis. Change-score analysis was used to examine how the change in LBM was affected by the changes in other covariates after 1 year of PD. The baseline LBM measurement was added into the multiple linear regression model of change-score analysis as a control covariate. The incidence of peritonitis was compared using Poisson analysis. Kaplan–Meier survival analysis and Cox regression proportional hazard analysis were used to analyze survival rates between groups and predictors for survival, respectively. p Values <0.05 were considered significant. The statistical analyses were performed using SPSS 13.0 for Windows (SPSS Inc., IL, USA).

## Results

### LBM in Patients on PD

A total of 115 incident PD patients were enrolled in the current study between January 2002 and December 2003 entered the current study. During the first year on PD, 12 patients dropped out (3 died, 3 underwent transplants, and 6 were transferred to HD). The remaining 103 patients were categorized into 2 groups according to median LBM values of 70% in men and 64% in women evaluated at 1 month after PD initiation. Group 1 included 52 patients with low LBM, and group 2 included 51 patients with high LBM. Clinical characteristics were compared between the 2 groups. Compared with group 2 patients, group 1 patients were older, had a higher incidence of diabetes mellitus (DM), and had lower serum albumin levels and nPCR (Table 1). The use of PD modality did not differ between these 2 groups.

**Table 1:** Comparison of clinical, nutritional, and clearance parameters between patients on peritoneal dialysis with low LBM (group 1) and high LBM (group 2).

	Group 1 (n = 52)	Group 2 (n = 51)
Age*	65±12	52±14
Men	24	25
DM*	14	1
APD	24	22
BMI (kg/m^2^)*	22.2±3.3	21.0±2.6
1Y BMI (kg/m^2^)*	23.8±3.6	22.0±2.6
nPCR (g/kg BW/day)*	1.06±0.24	1.16±0.20
1Y nPCR (g/kg BW/day)*	1.00±0.24	1.15±0.22
BUN (mg/dL)*	59±17	60±17
1Y BUN (mg/dL)	59±16	64±15
Creatinine (mg/dL)*	7.8±2.8	9.6±2.5
1Y Cre (mg/dL)	10.1±3.3	11.4±3.3
Albumin (g/dL)*	3.7±0.4	4.0±0.4
1Y albumin (g/dL)*	3.9±0.5	4.1±0.4
Cholesterol (mg/dL)*	191±53	216±48
1Y cholesterol (mg/dL)	213±51	222±44
Triglyceride (mg/dL)	154±116	132±59
1Y triglyceride (mg/dL)	229±147	191±163
TIBC*	221±56	245±43
1Y TIBC	240±57	251±31
Peritoneal Kt/V	1.74±0.37	1.76±0.36
1Y Peritoneal Kt/V	1.89±0.30	1.83±0.35
Renal Kt/V	0.56±0.43	0.68±0.40
1Y Renal Kt/V*	0.29±0.38	0.47±0.41
Total Kt/V	2.30±0.48	2.44±0.46
1Y Total Kt/V	2.18±0.37	2.30±0.36
Peritoneal WCCR (L/week)	38.4±8.5	40.0±7.4
1Y Peritoneal WCCR (L/week)	41.3±8.8	41.0±8.3
Renal WCCR (L/week)	31.9±23.8	32.5±20.7
1Y Renal WCCR (L/week)	15.2±17.5	21.7±20.3
Standard WCCR (L/week)	75.4±24.6	80.1±21.1
1Y standard WCCR (L/week)*	59.2±18.6	68.2±19.9
LBM (%)*	58±8	76±8
1Y LBM (%)*	59±11	74±12

* p<0.05 using Student’s *t* test for continuous variables or the Chi-square test for categorical variables.

1Y: the value evaluated 1 year after initiating peritoneal dialysis, APD: automated peritoneal dialysis with a cycler, BMI: body mass index, BUN: blood urea nitrogen, LBM: % of lean body mass corrected with body weight, nPCR: normalized protein catabolic rate, TIBC: total iron binding capacity, WCCR: weekly creatinine clearance.

### Predictors of LBM

The relationship between LBM and other nutritional and clinical parameters was further analyzed using Pearson correlation. LBM was negatively correlated with age, BMI, and fasting blood glucose levels and was positively correlated with BUN, creatinine, and albumin levels as well as TIBC and nPCR (Table 2). Peritoneal clearance and residual renal function were irrelevant to LBM.

**Table 2:** Correlation between LBM and other clinical characteristics in all patients on peritoneal dialysis.

	LBM	p
Age	−0.59	<0.001
BMI	−0.20	0.04
Renal KT/V	0.09	0.39
Peritoneal KT/V	−0.07	0.49
Total KT/V	0.02	0.81
Renal WCCR	0.02	0.80
Peritoneal WCCR	0.10	0.29
Standardized WCCR	0.08	0.40
4 Hr D/P Cre	0.09	0.36
BUN	0.33	<0.001
Creatinine	0.57	<0.001
Glucose	−0.47	<0.001
Albumin	0.37	<0.001
Cholesterol	0.13	0.20
Triglyceride	−0.11	0.26
TIBC	0.31	<0.01
nPCR	0.47	<0.001

BMI: body mass index, BUN: blood urea nitrogen, LBM: % of lean body mass corrected with body weight, nPCR: normalized protein catabolic rate, TIBC: total iron binding capacity, WCCR: weekly creatinine clearance.

From the above results, we concluded that nutrition was clearly associated with LBM. We further analyzed the independent determinants for LBM using multiple linear regression analysis. Age, DM, and BUN levels were negatively associated with LBM (Table 3). On the other hand, male gender, serum creatinine level, renal KT/V, and nPCR were positively associated with LBM. These independent determinants contributed to high predictability (R^2^ = 0.828; Table 3).

**Table 3:** Independent predictors for LBM with multiple linear regression analysis among patients on peritoneal dialysis.

	B±SE	p
Constant	23±6	<0.001
DM	−3.7±1.6	<0.05
Age	−0.2±0.1	<0.001
Men	6.4±1.2	<0.001
Creatinine	3.3±0.3	<0.001
Renal Kt/V	7.5±1.5	<0.001
nPCR (per 0.1 g/kg/day)	3.9±0.4	<0.001
BUN	−0.4±0.1	<0.001
R^2^	0.828	

BUN: blood urea nitrogen, LBM: % of lean body mass corrected with body weight, nPCR: normalized protein catabolic rate.

### LBM and Patient Outcomes

**Figure 1:**
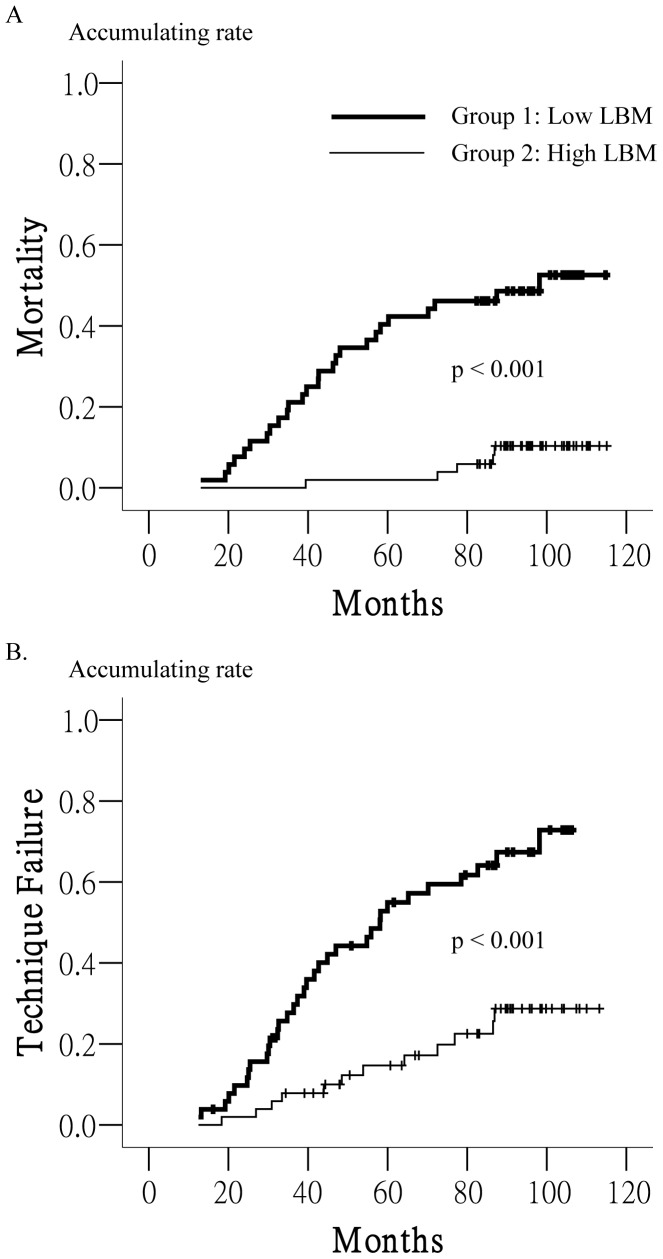
Lean Body Mass and Survival. Patients with low LBM (group 1) had shorter patient survival (A) and technique survival (B) than patients with high LBM (group 2) according to Kaplan–Meier survival analysis.

To clarify the impact of LBM on patient survival, the differences in outcomes between the 2 groups, including technique failure, mortality, and morbidity, were further analyzed. Both mean patient follow-up duration and time on PD were longer in group 2 patients (Table 4). The incidences of peritonitis and hospitalization and the duration of hospital stay were higher in group 1 patients. In addition, group 2 patients had a lower mortality rate, higher transplant rate, and higher PD duration. Kaplan–Meier survival analysis revealed that group 2 patients had longer patient (p<0.001, Figure 1) and technique survival (p<0.01, Figure 1).

**Table 4:** Comparison of variable target outcomes between patients on peritoneal dialysis with low LBM (group 1) and high LBM (group 2).

	Group 1 (n = 52)	Group 2 (n = 51)
Mean time on PD (M)*	57±30	75±26
Mean patient follow-up (M)*	71±31	95±12
Peritonitis/100 M*	1.6	1.1
Hospitalization days/100 M*	71	25
Hospitalization/100 M*	14.6	9.7
Outcome*		
Death	19	4
Transfer to HD	15	9
Transplant	4	15
PD	14	23

* p<0.05 using Student’s *t* test for continuous variables and Poisson analysis for incidence or Chi-square test for categorical variables.

Cox regression proportional hazard analysis was applied to determine the hazard ratio of LBM for mortality and technique failure. In univariate analysis, each percentage point increase in LBM reduced the hazard ratio for technique failure and mortality by 8% and 10%, respectively (Model 1; Tables 5 and 6). In multivariate analysis, LBM remained an independent factor for reducing the hazard ratio for technique failure, as did age and sex (Model 2, Table 5), even after controlling for DM and residual renal function (Model 3, Table 5). LBM also consistently reduced the hazard ratio for mortality after controlling for age and DM status (Model 2, Table 6), even after the addition of sex and residual renal function as variables (Model 3, Table 6). In Model 3, with control of several other variables, each percent increase in LBM was shown to reduce risk of mortality by 8%.

**Table 5:** Hazard ratio for dialysis technique failure according to Cox regression proportional hazard analysis using data at peritoneal dialysis initiation.

Technique failure			
Model 1	HR	95% CI for HR	p
LBM	0.92	0.89∼0.94	<0.001
Model 2			
LBM	0.93	0.91∼0.97	<0.001
Age	1.04	1.01∼1.07	0.01
Men	2.22	1.21∼4.08	0.01
Model 3			
LBM	0.94	0.91∼0.98	<0.01
Age	1.04	1.01∼1.07	0.02
Men	1.90	1.00∼3.62	0.05
DM	1.81	0.75∼4.35	0.19
Renal KtV	1.14	0.53∼2.47	0.74

CI: confidence interval, DM: diabetes mellitus, HR: hazard ratio, LBM: % of lean body mass corrected with body weight.

**Table 6:** Hazard ratio for mortality according to Cox regression proportional hazard analysis in all peritoneal dialysis patients.

Mortality			
Model 1	HR	95% CI for HR	p
LBM	0.90	0.87∼0.93	<0.001
Model 2			
LBM	0.93	0.89∼0.98	<0.01
DM	3.44	1.30∼9.10	0.01
Age	1.05	1.02∼1.09	<0.01
BMI	0.83	0.70∼1.00	0.05
BUN	1.05	1.00∼1.09	0.03
nPCR (per 0.1g/kg/day)	0.68	0.48∼0.97	0.03
Model 3			
LBM	0.92	0.88∼0.97	<0.01
DM	3.02	1.07∼8.48	0.04
Age	1.05	1.00∼1.09	0.03
BMI	0.82	0.68∼0.99	0.04
BUN	1.04	0.99∼1.09	0.11
nPCR (per 0.1g/Kg/day)	0.73	0.50∼1.1	0.11
Men	1.74	0.66∼4.55	0.26
Renal KtV	1.06	0.40∼2.87	0.90

BMI: body mass index, BUN: blood urea nitrogen, DM: diabetes mellitus, LBM: % of lean body mass corrected with body weight, nPCR: normalized protein catabolic rate.

### Factors Associated with Changes in LBM

Because LBM was shown to be important to the outcomes of patients on PD, the factors influencing LBM changes between the first month of PD treatment and 1 year later were further analyzed. Changes in LBM were inversely correlated with changes in BMI and directly correlated with changes in nPCR, creatinine levels, renal KT/V, and total KT/V at 1 year (Table 7). In change-score analysis with multiple linear regression analysis, nPCR, renal KT/V, creatinine levels, BUN, age, and baseline LBM changes were associated with changes in LBM with a high predictability of 0.712 (Table 8).

**Table 7:** Correlation between changes in LBM and changes in other parameters at 1 year.

	Changes in LBM	p
Age	−0.14	0.15
BMI	−0.25	0.01
nPCR	0.46	<0.001
Albumin	−0.04	0.69
TIBC	−0.07	0.49
Creatinine	0.44	<0.001
Glucose	−0.07	0.49
BUN	0.22	0.02
KtV	0.27	<0.01
Peritoneal KtV	0.17	0.08
Renal KtV	0.20	0.04

Change in each parameter is the change at 1 year divided by the initial value.

BMI: body mass index, BUN: blood urea nitrogen, LBM: % of lean body mass corrected with body weight, nPCR: normalized protein catabolic rate, TIBC: total iron binding capacity.

**Table 8:** Independent predictors for changes in LBM at 1 year of PD according to change-score analysis.

	B±SE	p
Constant	26.78±9.25	0.005
Change in nPCR	0.51±0.05	<0.001
Change in renal KtV	0.06±0.01	<0.001
Change in creatinine	0.33±0.03	<0.001
Change in BUN	−0.24±0.03	<0.001
Age	−0.23±0.07	0.002
LBM baseline	−0.24±0.08	0.006
R^2^	0.712	

Change in each parameter was the change at 1 year divided by the initial value.

BUN: blood urea nitrogen, LBM: % of lean body mass corrected with body weight, nPCR: normalized protein catabolic rate.

## Discussion

In this study, we followed a PD population for 8 years and showed that higher LBM predicts longer technique survival and overall patient survival. Changes in LBM were directly correlated with changes in nPCR, residual renal function, and serum creatinine and BUN levels. These results support that increasing protein intake and preserving residual renal function increase LBM, which would prolong patient survival.

High BMI is associated with increased survival in most patients on dialysis. However, this association cannot be applied to Asian patients on dialysis [14]. Different body components may have variable effects on the outcomes of patients on dialysis. LBM represents a non-fat component, but the role of LBM in this “reverse epidemiology” has remained unclear. Some authors have reported that LBM is not associated with this reverse outcome [14], but others have reported that LBM should be added to BMI to improve its predictive power [15]. Another study in patients on HD showed that increased fat mass reduced the hazard ratio for mortality, but LBM reduced mortality risk in women only and not in men [16]. Several studies showed that high LBM predicts a better outcome among patients on PD in short-term follow-up [5]–[7], [17]. In our study, which followed patients for up to 8 years, patients with low LBM had poorer survival and higher morbidity rates (Figure 1, Table 4). Incremental changes in both LBM and BMI reduced the hazard ratio for mortality, an effect that persisted after controlling for age, DM, and sex in Cox regression analysis (Table 6). Our findings further support that both LBM and BMI independently predict survival in patients on dialysis as demonstrated previously [15].

Serum albumin level is a well-known predictor for survival of patients on dialysis, although the relationships between LBM and albumin levels remain unclear. LBM represents somatic protein storage, while serum albumin level represents visceral protein. There was previously only a weak and even negative association between serum albumin and LBM in patients on HD [18]. In our study, LBM was positively correlated with serum creatinine and serum albumin levels in the PD population as shown previously [9]. In addition, LBM was also positively correlated with BUN and nPCR in the present study (Table 2). However, in multiple linear regression analysis, albumin lost its significance to predict LBM (Table 3). As a protein storage marker, LBM was not a substitute for albumin; rather, it provided a different representation of protein mass.

Because low LBM is a strong predictor of mortality in patients on PD, efforts to increase LBM should be valuable. Therefore, factors associated with LBM changes should be monitored to guide further management of patients on PD. Increased levels of physical activity and total daily protein intake are associated with higher LBM in patients on HD [18]. A study of patients on PD also showed that daily protein intake is positively correlated with LBM and associated with survival [17]. In our study, changes in LBM were positively correlated with changes in nPCR, creatinine, and renal KT/V and were negatively correlated with changes in BMI (Table 7). In other words, LBM increased as BMI decreased. Reducing body fat might reduce inflammation and increase LBM [18]. However, in patients on HD, higher fat mass is also associated with better survival [16], [19] as in the present study of patients with PD (Table 6; Models 2 and 3). This obesity paradox creates a dilemma for advising obese patients on dialysis to reduce body weight. Increased protein intake and preservation of renal function were both modifiable independent factors associated with increasing LBM and served as 2 main targets in caring for patients on PD.

LBM can be conveniently estimated using the equation for creatinine index in patients on PD because all variables represented in that equation are measured regularly in dialysis adequacy evaluation. The influence of overhydration and obesity are reduced in this method, which can provide a more reliable estimate of LBM than can other formulae [20]. Although the creatinine kinetics and PD dose shared similar variables, our results did not show any correlation between PD dose and LBM (Table 2). The association between change in residual renal function and in LBM might link to some pathogenesis caused by loss of renal function in the follow-up period.

An important strength of our study was its long follow-up period of 8–9 years. Its potential limitations include: First, the restricted case number and the lack of physical activity evaluation, which would be another factor for increasing LBM. Second, as seen in other observational studies, we cannot account for unmeasured or residual confounding variables. Finally, because our patients’ BMI values were 21–22 kg/m^2^, the confounding factor of obesity could not be adequately assessed.

In conclusion, low LBM is associated with higher mortality in patients on PD. Increasing protein intake and preserving residual renal function can increase LBM. Although these findings need to be confirmed by further studies, they may have important clinical implications regarding protein energy intake and weight reduction policy in patients on PD.
